# C-terminal fragments of the amyloid precursor protein in cerebrospinal fluid as potential biomarkers for Alzheimer disease

**DOI:** 10.1038/s41598-017-02841-7

**Published:** 2017-05-30

**Authors:** María-Salud García-Ayllón, Inmaculada Lopez-Font, Claudia P. Boix, Juan Fortea, Raquel Sánchez-Valle, Alberto Lleó, José-Luis Molinuevo, Henrik Zetterberg, Kaj Blennow, Javier Sáez-Valero

**Affiliations:** 1 Instituto de Neurociencias de Alicante, Universidad Miguel Hernández-CSIC, 03550 Sant Joan d’Alacant, Alicante España; 2 Centro de Investigación Biomédica en Red sobre Enfermedades Neurodegenerativas (CIBERNED), Sant Joan d’Alacant, 03550 Alicante España; 3 Unidad de Investigación, Hospital General Universitario de Elche, FISABIO, Elche Spain; 4 Memory Unit, Neurology Department, Hospital de la Santa Creu i Sant Pau, Barcelona, Spain; 5 Down Medical Center, Fundació Catalana Síndrome de Down, Barcelona, Spain; 6 Alzheimer’s Disease and Other Cognitive Disorders Unit, Neurology Service, Hospital Clinic, 08036 Barcelona, Spain; 7 Clinical Neurochemistry Laboratory, Sahlgrenska University Hospital, Mölndal, Sweden; 8 Institute of Neuroscience and Physiology, University of Gothenburg, Mölndal Campus, Sweden; 9 Department of Molecular Neuroscience, Institute of Neurology, University College London, London, UK

## Abstract

This study assesses whether C-terminal fragments (CTF) of the amyloid precursor protein (APP) are present in cerebrospinal fluid (CSF) and their potential as biomarkers for Alzheimer’s disease (AD). Immunoprecipitation and simultaneous assay by Western blotting using multiplex fluorescence imaging with specific antibodies against particular domains served to characterize CTFs of APP in human CSF. We demonstrate that APP-CTFs are detectable in human CSF, being the most abundant a 25-kDa fragment, probably resulting from proteolytic processing by η-secretase. The level of the 25-kDa APP-CTF was evaluated in three independent CSF sample sets of patients and controls. The CSF level of this 25-kDa CTF is higher in subjects with autosomal dominant AD linked to *PSEN1* mutations, in demented Down syndrome individuals and in sporadic AD subjects compared to age-matched controls. Our data suggest that APP-CTF could be a potential diagnostic biomarker for AD.

## Introduction

Accumulation of the β-amyloid peptide (Aβ) in the brain is an early and specific phenomenon associated with the pathogenesis of Alzheimer’s disease (AD)^1^. Many reports support that the determination of Aβ42 in cerebrospinal fluid (CSF) is a core biomarker for AD^2^. While the amount of the pathological species of Aβ is increased in the AD brain, their levels in CSF are decreased, probably due to increased brain deposition. To enable monitoring early disturbance in amyloid precursor protein (APP) and Aβ mis-metabolism additional biomarkers are needed.

Other plausible biomarkers for AD are additional fragments resulting from the processing of the amyloid precursor protein (APP). APP is a large type I transmembrane spanning protein consisting of a large N-terminal extracellular domain, a hydrophobic transmembrane domain, and a short intracellular C-terminal domain. APP is usually cleaved by α-secretase (ADAM10; leading to non-amyloidogenic pathway), or by β-secretase (BACE1; leading to amyloidogenic pathway), which causes the secretion of large sAPPα and sAPPβ N-terminal fragments (NTFs). CSF levels of sAPPα and sAPPβ show no change in AD^3^. The membrane remaining C-terminal fragments (CTFs) are always processed by γ-secretase generating shortest intracellular domain (AICD) peptides (for a review see Haass *et al*.^4^).

APP can also undergo alternative proteolytic processing pathways (for a review see Andrew *et al*.^5^). Thereby, concerted cleavage of β-secretase and α-secretase result in the secretion of sAPPβ and shorter Aβ peptides^6^. The presence in CSF of short NTF derivatives of APP, which generation does not involve α-secretase or BACE, has been also demonstrated^7^. More interestingly in the context of this report, a recently discovered alternative physiological APP processing pathway driven by an asparagine endopeptidase (AEP) named δ-secretase^8^ or by a metalloproteinase named η-secretase^9^ will generate alternative proteolytic metabolites, including NTFs and CTFs, of different molecular mass.

In addition to the different species of Aβ, all the large extracellular APP-NTFs have been studied in human CSF^9–11^; but the presence of APP-CTFs in CSF has not been reported to date. In this study we investigated if APP-CTFs are detectable in CSF, characterized the major APP-CTF immunoreactive band, and determined whether the levels of this peptide fragment are altered in autosomal dominant AD (ADAD), Down syndrome subjects with Alzheimer’s type dementia (dDS), and sporadic AD subjects (sAD).

## Results

### APP-CTFs are present in human CSF

**Figure 1:**
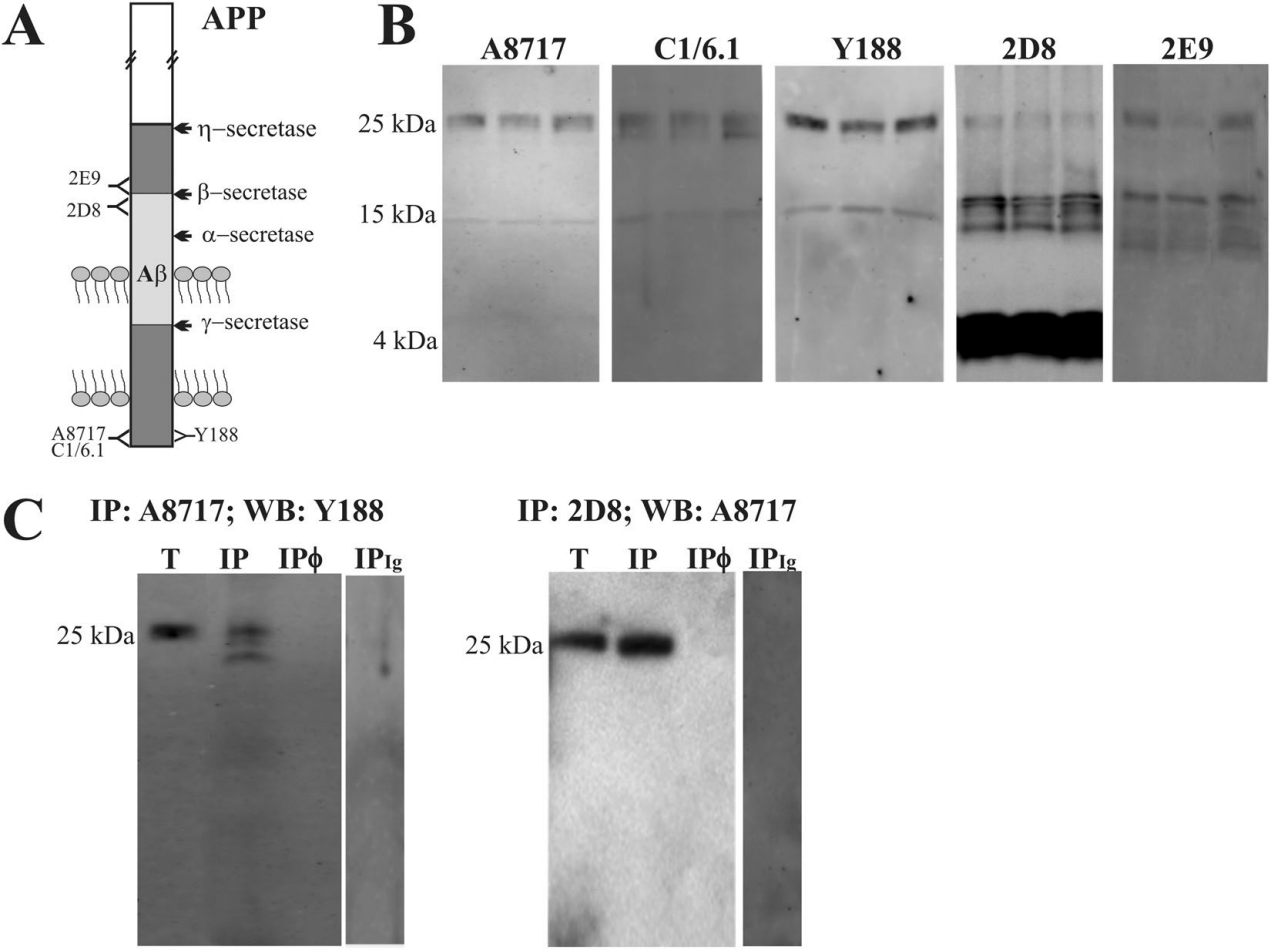
Soluble CTFs of APP are present in human CSF. (**A**) Schematic representation of full-length APP processing by secretases. The epitopes for the antibodies used in this study are indicated. (**B**) Western blotting of three human CSF samples from non-demented controls subjects, resolved with the indicated antibody (samples were the same between different blots). (**C**) CSF aliquots (Total, T) were immunoprecipitated with the indicated antibody, and precipitated proteins (bound fraction, IP) were immunoblotted with the indicated alternative antibody. CSF aliquots incubated with protein A-Sepharose in the absence of capture-antibody (IPø) or with an irrelevant IgG (IP_Ig_; a rabbit IgG for A8717 IP and a rat IgG for 2D8 IP), were analyzed in parallel as negative controls.

To determine the presence of APP-CTFs in human CSF, we first examined human CSF samples by Western blotting using three different anti-CTF antibodies (a schematic representation of APP and epitopes for antibodies is represented in Fig. 1A). This three APP-CTF antibodies demonstrated specificity in assess the accumulation of C-terminal fragments of APP in cells transfected with a construct that encodes the C-terminal 99 amino acids of APP (amino acids 597–695; blots not shown). Immunoblotting revealed a similar pattern of immunoreactive bands, with a predominant band of ~25 kDa (Fig. 1B). This band was also observed with the 2D8 and 2E9 antibodies, both against an extracellular domain close to the transmembrane domain, thus recognizing a η-secretase-generated CTF-APP (CTFη)^9^. 2D8 and 2E9 also detected other soluble fragments around ~15 kDa, termed Aη, generated after concerted cleavage by η-secretase and α/β-secretases^9^. 2D8 antibody also detected Aβ monomers. Multiplex fluorescence imaging with the A8717 and 2E9 antibodies indicated that the major 25 kDa band is compatible with the CTFη; similar co-labelling was revealed with antibodies C1/6.1 and 2D8 (Supplemental Fig. 1). To further examine the identity of CTF bands in human CSF, we performed immunoprecipitation/Western blot analysis (Fig. 1C). Since in SDS-PAGE the immunoglobulin light chain migrates at similar molecular mass that the APP-CTF 25-kDa band, we used dimethyl pimelimidate dihydrochloride to covalently link primary antibodies to protein A Sepharose, and eluted with 0.1 M glycine buffer at pH 2.5, in order to prevent the co-elution of the antibody along with the target protein^12^. CSF samples were immunoprecipitated using the A8717 antibody and blotted with the Y188 antibody, confirming the identity of the 25-kDa band as an APP-CTF. Immunoprecipitating with 2D8 antibody and blotting with A8717 antibody supported the identity of the 25-kDa band as a CTFη.

### The 25-kDa APP-CTF is increased in AD CSF

**Figure 2:**
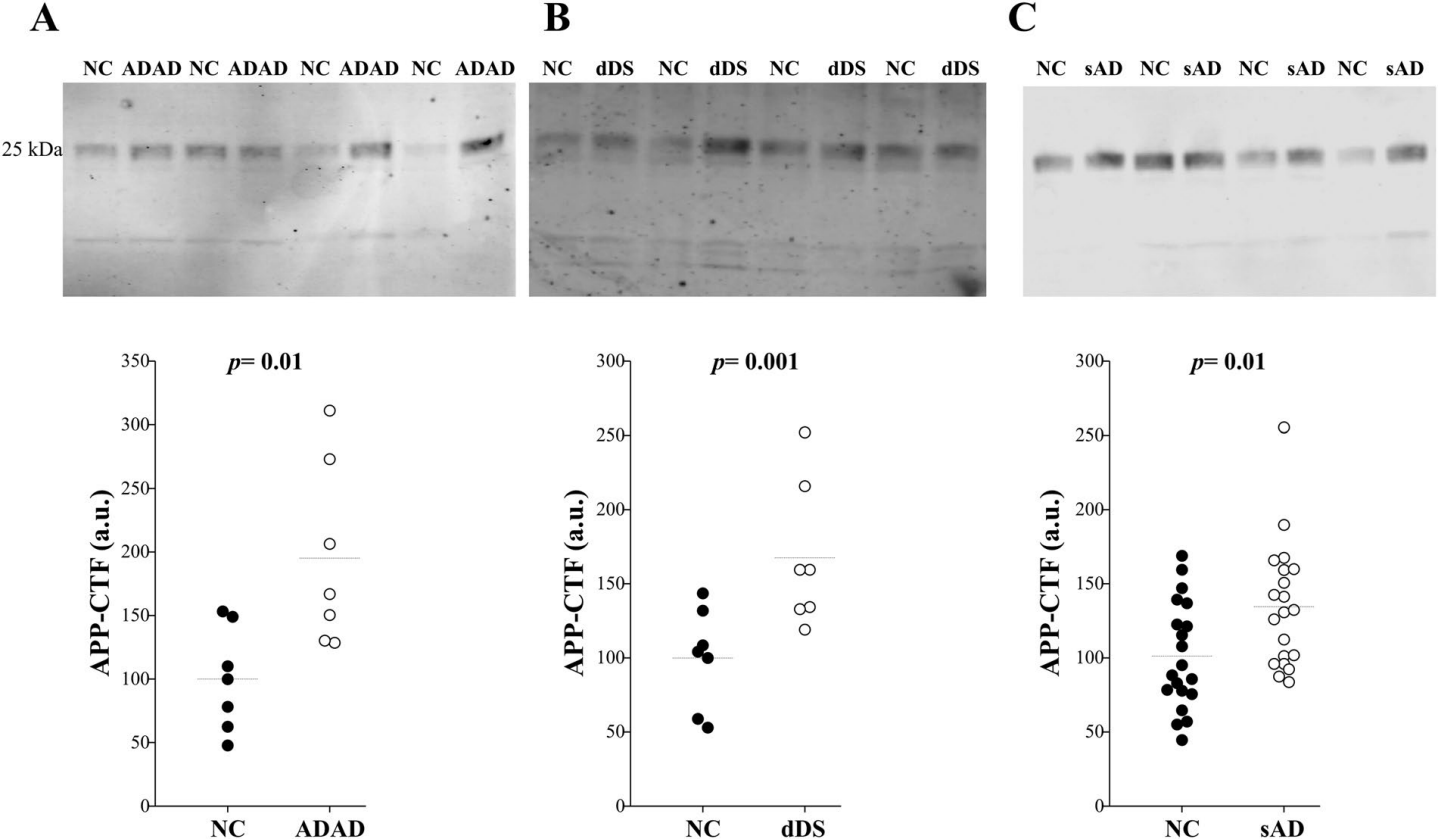
Higher levels of the 25-kDa APP-CTF band in the CSF of AD subjects. (**A**) Representative blot of the 25-kDa APP-CTF in the CSF samples from 7 symptomatic ADAD and 7 age-matched NC, which were from the same families that ADAD subjects but that did not carry mutations (black symbol; see also Table 1). Densitometric quantification of the immunoreactivity from the 25-kDa band is also shown. (**B**) Representative blot and densitometric quantification in CSF from 7 DS subjects with dementia of the Alzheimer’s type (dDS) and 7 age-matched NC. (**C**) Representative blot and densitometric quantification of the immunoreactivity from the 25-KDa APP-CTF in CSF samples from 20 sAD and 20 age-matched NC subjects. Immunodetections for (**A**, **B**, **C**) were performed with the A8717 antibody. *p* values are displayed.

To assess whether APP-CTF levels are altered in AD, we first analyzed CSF samples from autosomal dominant AD (ADAD), an early-onset form of genetically determined AD^13^. Clinical, demographic data and classic CSF biomarker levels are included in Table 1. Genetically determined AD offers unique opportunities to analyze diagnostic biomarkers particularly given that diagnosis is guaranteed. The 25-kDa band was detected with A8717 antibody in all CSF analyzed (Fig. 2A). The immunoreactivity of the 25-kDa APP-CTF band in the CSF from ADAD subjects increased (95 ± 27%; *p* = 0.01) compared to those in age-matched NC composed by non-mutation carriers from the same families (Fig. 2A). Down syndrome subjects with Alzheimer’s type dementia (dDS) can be also considered a form of genetically determined AD^13^. Once more, an increase (68 ± 18%; *p* = 0.01) in the intensity of the 25-kDa APP-CTF band, resolved with A8717 antibody, was determined in CSF samples from dDS patients, comparing these to age-matched NC (Fig. 2B). Finally, we also have assessed potential differences in the level of the 25-kDa APP-CTF band in sporadic AD subjects (sAD) compared to aged-matched controls (see Table 1). We found that the total immunoreactivity for this band, detected with the A8717 antibody, increased (35 ± 9%; *p* = 0.01) in sAD compared to age-matched NC subjects (Fig. 2C). CSF samples from sAD subjects also showed a similar increment for the 25-kDa band when they were analyzed with the 2D8 antibody (35 ± 9%; *p* = 0.01), correlating tightly with the estimation of the levels calculated with the A8717 antibody (R = 0.73; *p* < 0.001; Supplemental Fig. 2). There were no clear correlations between the level of the 25-kDa APP-CTF band and the levels of classical biomarkers, Aβ42, T-tau or P-tau, in either the control or sAD groups considered individually (Supplemental Fig. 3). Only when control and sAD subjects were pooled, a positive correlation emerged between immunoreactivity levels of the 25-kDa APP-CTF band with T-tau, and with P-tau levels (Supplemental Fig. 3).

**Table 1:** Clinical, demographic data and classic CSF biomarker levels.

Group	Age (years)	Gender	CSF Aβ42 (pg/mL)	CSF T-tau (pg/mL)	CSF P-tau (pg/mL)
sAD	71 ± 2 [55–86]	15F/5M	412 ± 19**	665 ± 52**	84 ± 6**
NC	72 ± 2 [57–88]	6F/14M	739 ± 32	233 ± 14	36 ± 2
ADAD	43 ± 2 [31–49]	5F/2M	266 ± 49**	883 ± 204**	168 ± 69*
NC	39 ± 3 [25–47]	5F/2M	809 ± 94	245 ± 29	46 ± 4
dDS	53 ± 2 [43–57]	4F/3M	422 ± 17*	767 ± 170*	108 ± 19*
NC	48 ± 2 [47–53]	5F/2M	751 ± 84*	160 ± 26	33 ± 5

The *PSEN1* mutations included in this study from ADAD cases (autosomal dominant AD subjects) corresponded to 3 carriers of L286P, and one S169P, L173F, L235R and L282R. The data represent the means ± SEM, and for age the range of values is also indicated. All the pathological groups were compared with age-matched NC obtained from the same Hospital. Significantly different **p < 0.005; *p < 0.05 from the NC group.

## Discussion

Here, we demonstrate that APP-CTFs are detectable in human CSF. Particularly, the most abundant soluble APP-CTF, a 25-kDa fragment, is attributed to a CTF recently characterize as the result of proteolytic processing by η-secretase (CTFη)^9^. Canonical APP proteolysis occurs via α- and β-secretases, resulting in CTFα and CTFβ with a molecular mass lower than 15 kDa; but novel APP-CTF that migrates ~25 kDa has also been described^14^. In addition to the described alternative pathways for APP^7–9^, other cleavages for APP could occur since non-canonical N-terminal APP fragments have been also identified in human CSF^15^. The precise characterization of the APP-CTF 25-kDa fragment as a CTFη requires further confirmation. In this regard, the development of pan-specific antibodies against the predicted N-terminus of CTFη could be valuable. Similarly to the reported by others^15^, attempts to immunoprecipitate the 25-kDa APP-CTF for sequencing were not fruitful. Since soluble full-length APP protein is also present in CSF, forming heteromers that co-immunoprecipitate with more abundant sAPPβ and sAPPα^16^, an enrichment process based in available APP C-terminal antibodies could result of lower efficient and specificity for APP-CTFs. Similar handicap will difficult development of a specific ELISA assay (discussed below).

As an intermediate of proteolytic process, the presence of any APP-CTF in CSF was unexpected. Anyhow, the presence in CSF of soluble forms of full-length membrane proteins containing the transmembrane and intracellular domains is not an unusual finding^16, 17^. Early studies failed to detect short C-terminal fragments of APP in the medium of cultured cells^18^; but others suggested the release of largest APP-CTF fragments from cultured neural cells^19^. Moreover, a series of APP-CTFs was found to be secreted within exosomes in cultured cells^20^, as well AICD^21^. The mechanisms by which these membrane-bound proteins reached the CSF are unknown; but neuronal death may be also a major contributing factor. Regarding the cellular source of the soluble 25-kDa APP-CTF found in CSF, the presence of hitherto undocumented APP fragments, including a 25-kDa CTF, has been documented in hippocampal neurons^9^, but also across other multiple cell types, including bovine brain microvascular endothelial cells^15^. Since the δ-secretase enzyme AEP is a widely distributed^22^, we could not discard a non-neural source for the 25-kDa APP-CTF found in CSF. The cellular origin of the 25-kDa APP-CTF could be also relevant in order to define their potential as an AD biomarker.

Different APP C-terminal antibodies confirmed the 25 kDa as the predominant band in human CSF. As stated, these APP proteolytic fragments are derived from a constitutive processing step driven by a η-secretase that is partially in dynamic equilibrium with α- and β-secretase-mediated proteolysis^9, 23, 24^. The current canonical views about APP-related cleavage events are likely a partial understanding of the overall process. How secretases compete for the APP substrate and whether subcellular compartmentalization of APP and secretases is responsible of the dynamic equilibrium between secretases is under discussion^25^. Thus, one may speculate that the particular abundance of the 25-kDa CTFη in CSF is related with compartmentalization of secretases, resulting in prolonged time of residence of these CTFη in comparison with canonical CTFα and CTFβ. Anyhow, the other fragments derived of the further processing of CTFη by α- and β-secretases, the Aη-α and Aη-β fragments, have been also identified in human CSF with levels similar than those for Aβ^9^.

Our determination of the 25-kDa APP-CTF levels by Western blotting displayed overlapping values between sAD and controls, whereas superior discrimination was obtained for ADAD and dDS subjects, compared to their respective controls. Interestingly, APP CTFβ levels are also elevated in frontal cortex brain homogenates obtained from ADAD patients^26^. Further studies will indicate if distinct pattern of APP processing in ADAD and sAD affect the generation of different APP-CTFs. Given that this is the first report that address the levels of APP-CTF in CSF, in order to prevent influence of pre-analytical confounding factors, in our study each pathological group was compared with age-matched controls obtained from the same center, with same volume of CSF taken, stored in aliquot of same volume and for similar period of time; and avoiding freeze/thaw cycles. Further studies are required to determine whether limiting pre-analytical confounding factors (discussed in del Campo *et al*.^27^), have a significant impact on the measured levels of APP-CTF by Western blotting. The possibility that APP-CTF levels varies with age should also be considered and deserve a specific study with a larger number of samples.

In our statistical analysis high levels of the 25-kDa APP-CTF in CSF correlated with increased concentration of T-tau and P-tau, but failed to correlate with Aβ42 levels. Increased concentration of T-tau in CSF is believed to reflect neurodegeneration whereas P-tau is supposed to reflect tangle pathology. Otherwise, the overall decrease in Aβ42 concentration in the CSF patients is not illustrative of imbalances in the brain APP processing, since in parallel with the increase in the generation of Aβ42, there will be an increment in Aβ aggregation with subsequent deposition. Thus, the positive correlations of the 25-kDa APP-CTF with T-tau and P-tau suggest a correlation with AD pathological process, but it is not possible to interpret links with altered APP processing. Anyhow, the mentioned correlations resulted positive only when all samples were considered, and not in control or sAD groups considered separately. Thus, these correlations obtained by pooling controls and demented subject, could be artefactual, driven by the anchoring effect of the control values. Further analysis with a large number of samples is required.

In term to define the true potential of the 25-kDa APP-CTF as a CSF biomarker, it will be valuable to replicate our present finding using techniques such as ELISA. However, this desirable outcome will be challenging. As advanced above, we have described that the soluble full-length APP, containing the C-terminal domain, also exists in CSF as heteromeric complexes compromising other sAPP species^16^, and transmembrane domain, as well intracellular C-terminal domain could participate in APP dimerization (discussed in Isbertet *et al*.^28^). Indeed, intracellular CTF aggregates have been described with the participation of Aβ^29^ and heparan sulfate degradation products^30^, suggesting that other APP fragments and other proteolytic sub-products could interact with APP-CTFs. Thus, future studies should develop custom pan-specific antibodies targeting the predicted N-terminal sequence of the APP-CTFs present in CSF, maybe including pretreatment methods designed to disaggregate peptides^31^, for preventing co-precipitation of other APP fragments. Anyhow, even the disadvantages of Western blotting for quantitative analysis, we considered demonstrated that accumulation of APP-CTFs in CSF constitute a potential new biomarker of AD.

In resume, to our knowledge, the possibility that APP-CTFs can be assessed in the CSF has thus far not been considered. In this study, we demonstrate noticeable amounts of APP-CTFs in human CSF. Particularly, a 25-kDa APP-CTF appears increased in genetically determined AD, as well as in sAD. Our present findings provide sufficient evidence to justify further studies on the determination of APP-CTFs as a potential new diagnostic biomarker of AD.

## Material and Methods

### Patients

Lumbar CSF samples were obtained from autosomal dominant AD (ADAD) subjects that were all carriers of *PSEN1* mutations and who were part of the Genetic Counseling Program for familiar dementia (PICOGEN) at the Hospital Clínic (Barcelona, Spain). This group included 7 subjects carrying *PSEN1* mutations, and 7 age-matched non-mutation carriers from the same families (non-disease controls: NC). We also included lumbar CSF samples from 7 Down syndrome subjects with Alzheimer’s type dementia (dDS), along with 7 age-matched NC obtained from the Hospital Sant Pau (Barcelona, Spain). In addition, 20 subjects with sporadic AD (sAD) defined as patients with cognitive symptoms and a CSF biomarker profile indicating AD (high total tau and phosphorylated tau together with low Aβ42 levels; see Table 1) and 20 age-matched controls defined as patients with non-specific symptoms without neurochemical evidence of AD were also obtained from the Clinical Neurochemistry Laboratory (Mölndal, Sweden). All AD patients fulfilled the 2011 NIA-AA criteria for dementia^32^. This study was approved by the ethic committee at the Miguel Hernandez University and it was carried out in accordance with the Declaration of Helsinki. All patients (or their nearest relatives) and controls gave informed consent to participate in the study.

### Western blotting and immunoprecipitation

Samples of CSF (30 µL) were denatured at 98 °C for 5 min and were resolved by electrophoresis on 16.5% Tris-Tricine gels. Following electrophoresis, proteins were blotted onto nitrocellulose membranes (Schleicher & Schuell Bioscience, GmbH, Dassel, Germany). CTFs of APP were detected using the following anti-APP C-terminal antibodies: C1/6.1 (mouse monoclonal; Covance Inc, Princeton, USA), A8717 (rabbit polyclonal; Sigma Aldrich Co, St. Louis, MO), and Y188 (rabbit monoclonal; Abcam, Cambridge, UK), as well as with the rat antibodies 2D8 and 2E9^9^. Multiplex fluorescence with two of the independent antibodies served to assess the specificity of the bands. Blots were then probed with the appropriate conjugated secondary antibodies (IRDye 680RD goat anti-rabbit; IRDye 680RD goat anti-mouse; and IRDye 800CW goat anti-rat from LI-COR Biosciences Lincoln) and imaged on an Odyssey Clx Infrared Imaging System (LI-COR Bioscences). Band intensities were analyzed using LI-COR software (Image Studio Lite). A control CSF sample was used to normalize the immunoreactive signal between blots.

For immunoprecipitation, samples were precleared for 2 h at 4 °C by incubation with protein A-Sepharose (Sigma Aldrich Co). Immunoprecipitations were performed overnight at 4 °C by incubating 150 µL of CSF with the indicated anti-APP antibody. The antibody was previously coupled to protein A-Sepharose using dimethyl pimelimidate dihydrochloride (Sigma Aldrich Co). A rabbit IgG and a rat IgG (Vector) were used as control antibodies for immunoprecipitations. Precipitated proteins were washed with PBS and eluted with 0.1 M glycine buffer at pH 2.5. After pH neutralization, supernatants were denatured in Laemmli sample buffer at 98 °C for 5 min and subjected to SDS-PAGE. The membranes were then probed with an alternative anti-APP antibody.

### Measurement of T-tau, P-tau and Aβ42 by ELISA

Total tau (T-tau), phosphorylated tau (P-tau) and Aβ1–42 (Aβ42) concentrations in CSF were measured using INNOTEST ELISA methods (Fujirebio Europe, Gent, Belgium).

### Statistical analysis

All data were analyzed using SigmaStat (Version 3.5; Systac Software Inc.) by Student’s *t* test (two-tailed) for determination of exact *p* values. Correlation was assessed by linear regression. Results are presented as means ± SEM.

## Electronic supplementary material

Supplemental figures
