# Supplementary material for: C-terminal fragments of the amyloid precursor protein in cerebrospinal fluid as potential biomarkers for Alzheimer disease
Source: Sci Rep. 2017 May 30;7:2477. doi: 10.1038/s41598-017-02841-7 (PMC5449401; doi:10.1038/s41598-017-02841-7)
Supplement: Supplementary file 1 — Supplemental figures [file 41598_2017_2841_MOESM1_ESM.pdf]

Supplementary Figures

C-terminal fragments of the amyloid precursor protein in cerebrospinal fluid as potential biomarkers for Alzheimer disease.

María-Salud García-Ayllón[1,2,3]; Inmaculada Lopez-Font[1,2]; Claudia P. Boix[1,2]; Juan Fortea[2,4,5]; Raquel Sánchez-Valle[6]; Alberto Lleó[2,4]; José-Luis Molinuevo[6]; Henrik Zetterberg[7,8,9]; Kaj Blennow[7,8]; Javier Sáez-Valero[1,2,*].

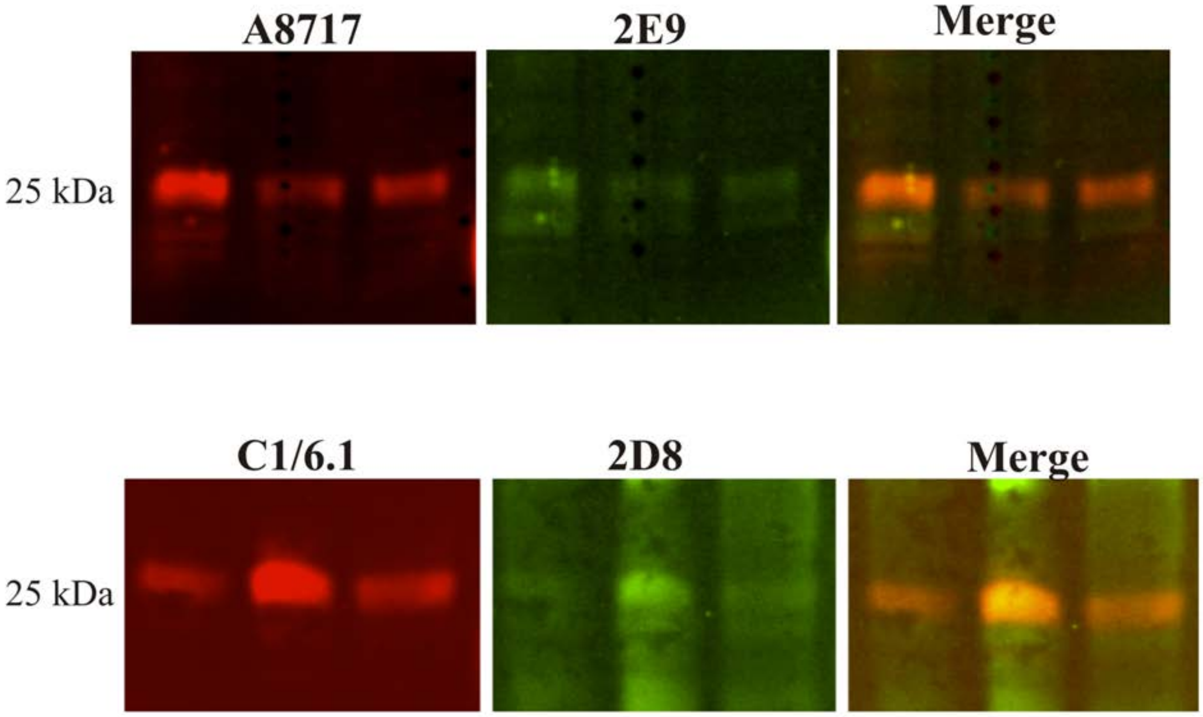

***Supplemental Figure 1*. SDS-PAGE analysis and fluorescence detection of soluble CTFs of APP in human CSF**. To probe that the 25-kDa specie resolved with different antibodies is the same, three aliquots of CSF were analyzed by SDS-PAGE and resolved with (**A**) A8717 and 2E9 antibodies, or (**B**) C1/6.1 and 2D8 antibodies, simultaneously. The fluorescence of the secondary antibodies was detected with the Odyssey CLx Infrared Imaging system (LI-COR).

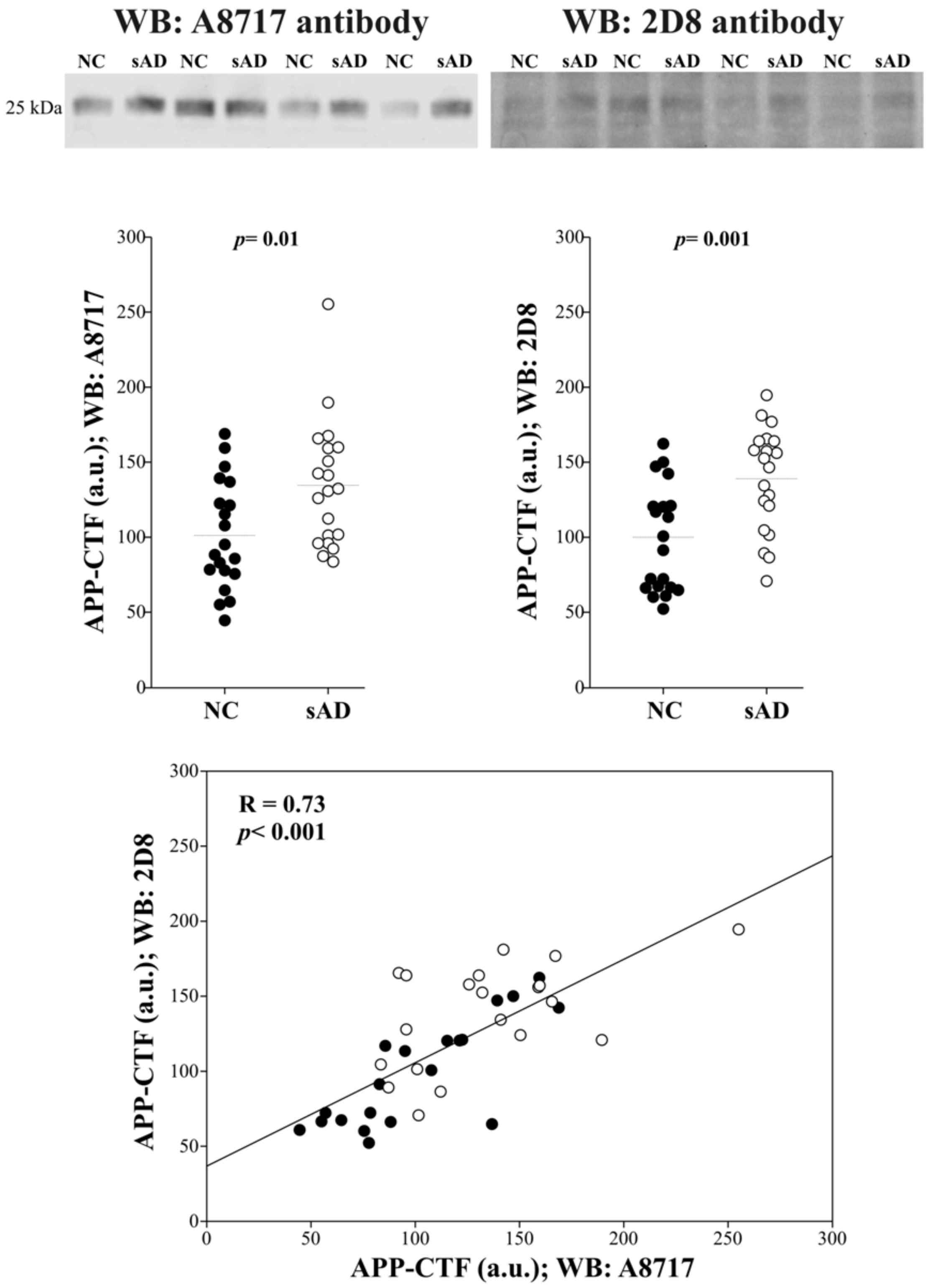

Supplemental Figure 2. **Corroboration of the increase in the 25-kDa APP-CTF in the CSF of sAD subjects.** Representative blot and densitometric quantification

of the 25-kDa APP-CTF in the CSF samples from 20 probable sAD 20 age-matched NC subjects blotted with the A8717 antibody (same data that in Fig. 2) and with the 2D8 antibody. A positive correlation was obtained when immunoreactive levels estimated with both antibodies from the same subjects were confronted. Regression linear coefficient (R) and *p* values are displayed.

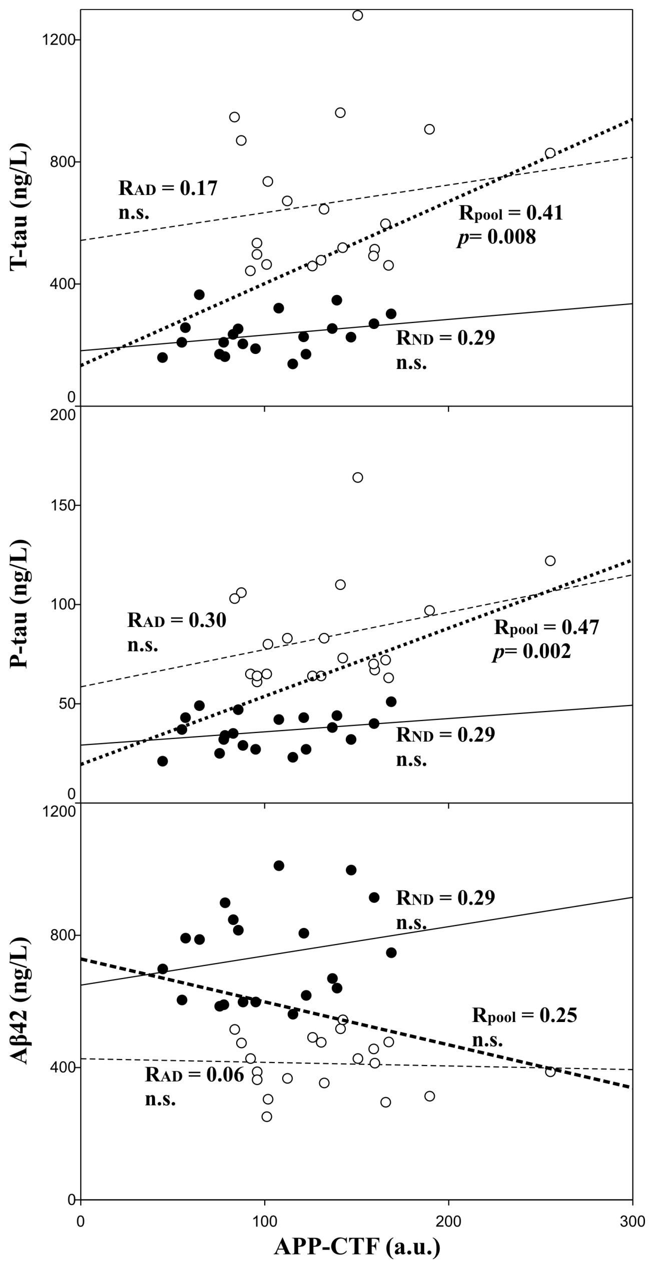

Supplemental Figure 3. Correlation of the 25-kDa APP-CTF with Aβ42, T-tau and P-tau levels in CSF samples. A linear regression was used to assess the correlation between the immunoreactivity level of the 25-kDa APP-CTF (Fig. 2) and Aβ42, T-tau and P-tau levels obtained by ELISA (see Table 1), in the samples from age-matched cognitively normal controls (NC: closed symbol, solid lines) and sAD patients (open symbol, dotted lines). The linear regressions resulted by pooling NC and sAD samples are also showed (thickened lines). The linear regression coefficient (R) and *p* values for each correlation are shown (n.s.: non-significant *p* value).
